# Novel *crystallin gamma B* mutations in a Kuwaiti family with autosomal dominant congenital cataracts reveal genetic and clinical heterogeneity

**Published:** 2012-12-09

**Authors:** Suad AlFadhli, Sidky Abdelmoaty, Amal Al-Hajeri, Abdulmutalib Behbehani, Fowzan Alkuraya

**Affiliations:** 1 Department of Medical Laboratory Sciences, Faculty of Allied Health Sciences, Kuwait University, Kuwait; 2 Al Bahar Eye Centre, Ministry of Health, Kuwait; 3 Department of surgery, Faculty of Medicine, Kuwait University, Kuwait; 4 College of Medicine -AlFaisal University, Department of Genetics, King Faisal Specialist Hospital and Research Centre, Riyadh, Saudi Arabia

## Abstract

**Purpose:**

To explore the disease locus and causative mutation for autosomal dominant congenital cataracts (ADCC) in a Kuwaiti family. There were seven affected and three unaffected subjects in the family.

**Methods:**

Whole-genome linkage analysis was performed using Gene Chip Human Mapping 250 K Arrays to identify regions of linkage. Potential genes within this region were cloned and sequenced to identify the disease-causing mutation.

**Results:**

The highest logarithm of odds score (1.5) region 2q34–36.1, spanning the crystallin beta A2 (*CRYBA2*) gene, showed no sequence changes. Thus, the second highest logarithm of odds score (1.49) region, 2q33–37, spanning the gamma crystalline gene cluster (*CRYG*), was considered. Sequencing of the *CRYGA*, *B*, *C*, and *D* genes revealed two novel heterozygous deletions and one trinucleotide polymorphism in the *CRYGB* gene. These mutations included a heterozygous g.67delG, intron 1 deletion in four of the affected family members with lamellar cataracts and a heterozygous g.167delC, exon 2 deletion inherited from the Egyptian grandmother by her granddaughter, resulting in anterior polar cataracts. Another patient with complete cataracts was a compound heterozygote with both of the above-mentioned mutations. In addition, the novel trinucleotide polymorphism g.20–22 GGT>AAA was detected in three of the family members.

**Conclusions:**

We report the linkage of ADCC to chromosome 2q33–37, which spans the *CRYGB* gene. This study is the first to report complex heterogeneous mutations in the *CRYGB* gene resulting in ADCC with three distinct phenotypes (lamellar, anterior polar, and complete cataracts) in the same family.

## Introduction

Cataract, an opacification of the lens of the eye, is a major cause of blindness worldwide, and can be broadly divided into early onset (congenital or juvenile) and age-related cataracts. Congenital cataract is a clinically and genetically heterogeneous lens disorder that is responsible for approximately one-tenth of childhood blindness worldwide. The prevention of visual impairment due to congenital cataracts is an important component of the World Health Organization’s international program for the elimination of avoidable blindness by 2020 [1].

Studies on hereditary congenital cataracts have led to the identification of genes involved in the formation of cataracts. Most inherited cataracts are caused by mutations in genes that encode eye lens proteins. Lens transparency is suggested to rely on a set of water-soluble proteins called crystallins [2,3]. High concentrations of closely packed crystallins are required for lens transparency and the ability to focus light on the retina. Together, the three major types of crystallins—alpha (α), beta (β), and gamma (γ)—comprise approximately 90% of lens proteins [4]. The α crystallins, which consist of the αA and αB subunits, are members of the small heat shock protein family, and have a chaperone-like activity that prevents the aggregation of denatured proteins [5-7]. The β and γ crystallins are structurally related and consist of four similarly folded Greek key motifs organized into two domains. Because of their high levels of expression, crystallins represent compelling candidate genes for inherited cataracts.

Different mutations in crystallin genes could lead to distinctive cataract phenotypes. To date, 13 distinct loci in humans have been identified to be responsible for 10 phenotypically distinct forms of autosomal dominant congenital cataracts (ADCC) [8]. A broad variety of mutations responsible for congenital cataracts has been discovered in recent years, including mutant forms of α crystallin [9,10], βA3/A1 crystallin [11,12] βB1 crystallin [13], βB2 crystallin [14,15], γC crystallin [16,17], and γD crystalline [18]. Nevertheless, a long list of mutations remains to be characterized and functionally investigated. This has encouraged us to search for the disease locus associated with ADCC in three generations of a Kuwaiti family. Two novel heterozygous mutations in the crystallin gamma B (*CRYGB*) gene that cause autosomal congenital lamellar, anterior polar, and complete cataracts were detected in this study. In addition, a novel maternally inherited trinucleotide polymorphism was found. This study is the first to report a mutation in the human *CRYGB* gene.

## Methods

### Family data

**Figure 1:**
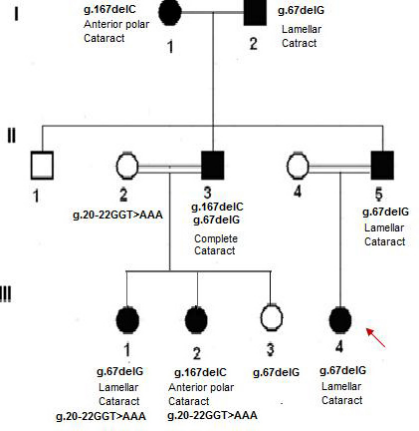
Pedigree of Kuwaiti family affected by autosomal dominant congenital cataract. Two novel mutations in the crystalline gamma B (*CRYGB*) gene resulting in three distinct phenotypes were detected. In addition, the novel tri nucleotide polymorphism was detected in three of the family members. The genotype and the resulting phenotype of each affected member are shown. The arrow indicates the proband.

This study aimed to explore the disease locus responsible for ADCC in three generations of a Kuwaiti family in which the grandmother was originally from Egypt. The family consists of seven affected and three unaffected subjects. Four of the affected family members were diagnosed with lamellar cataracts, while two others have anterior polar cataracts and one has complete cataracts. The patients affected by lamellar, anterior polar, and complete cataracts received clinical and ophthalmological examinations. There was no history of other ocular or systemic abnormalities in this family. Fifty unrelated age-, gender-, and ethnically matched healthy volunteers were recruited for this study. Ethnic bias within the subjects studied was minimized by excluding patients who were not of Kuwaiti origin. An autosomal dominant mode of inheritance was indicated by the pedigree (Figure 1). The study was approved by the institution’s ethics committee, and all of the participants provided written informed consent.

### Extraction of DNA from peripheral blood

Blood samples were collected from all of the affected and unaffected family members. Genomic DNA was extracted using a QIAamp Blood DNA mini kit (Qiagen, Germany) and quantified spectrophotometrically using a NanoDrop Spectrophotometer (ND-1000). PCR was performed using AmpliTaq Gold ready-to-use Master Mix (Applied Biosystems, Foster City, CA). Specific primers were used to amplify the coding and noncoding regions of the *CRYGBA2* and *CRYG(A-D)* gene cluster (sequences available upon request). The thermal amplification program consisted of an initial denaturation step (10 min at 95 °C), followed by 35 cycles of 1 min denaturation (95 °C), 1 min annealing (58 °C), and 1 min elongation (72 °C), with a final extension period of 7 min at 72 °C. The annealing temperature varied for each set of primers. PCR products were separated on 2% agarose gels and detected by transillumination with UVI-Prosoft.

### Genome-wide linkage analysis

A high-density single-nucleotide polymorphic (SNP) genome scan was performed using a Gene Chip Human Mapping 250 K Array, version 2 (Affymetrix, Santa Clara, CA). This whole-genome sampling array comprises 250,000 SNP markers with an average intermarker distance of 0.2 cM. The analysis was performed according to the manufacturer’s protocol (Assay Manual 2006, Affymetrix Inc.). Parametric multipoint linkage analyses were performed using Allegro v1.2c (Decode Genetics, Iceland) from the easy linkage Plus version 5.02 package (University of Wurzburg, Germany), and errors were removed using Pedcheck software version 1.1. The SNP marker allele frequencies used for linkage analysis were those calculated for Caucasians by the HapMap project (ref. 2003). Autosomal dominant inheritance with complete penetrance and a disease gene frequency of 0.0001 were assumed. Recombination values (θ) were considered to be equal in males and females. Computation was performed with Allegro in sets of 100 markers. The genetic positions of nonparametric linkage and logarithm of odds (LOD) scores for all chromosomes were indicated in relation to the shifting level model (SLM) map.

### Cloning and sequencing of DNA

Genomic DNA samples from all of the affected and unaffected family members in the pedigree were screened for mutations. *CRYBA2, CRYGA*, *CRYGB*, *CRYGC*, and *CRYGD* were cloned into the pGEM TA cloning vector (Promega, Madison, WI) and directly sequenced. Sequencing PCR was performed using gene-specific primers (sequences available upon request) and the BigDye terminator FS Ready Reaction Kit (Applied Biosystems). Reaction products were purified using the sodium acetate method and sequenced with an ABI PRISM 3100 DNA sequencer (Applied Biosystems).

## Results

This study was aimed at investigating the phenotypic heterogeneity and causative mutations of ADCC in a Kuwaiti family across three generations using whole-genome linkage analysis. A region of potential linkage with a maximum LOD score of 1.5 was identified on chromosome 2q34–36.1. This region spans 7.6 mega base pairs (Mb) of genomic DNA and includes 98 genes. The crystallin βA2 (*CRYBA2*) gene in this region was considered to be a candidate gene for cataractous mutation based on its known function. However, no sequence alteration was found in the coding or noncoding regions of affected family members, thus excluding its association with ADCC in the present family. Therefore, we considered the second highest LOD score (1.49) region on chromosome 2q33-q37, which spans the ɣ crystallin gene cluster (A-D). All four genes—*CRYGA*, *CRYGB*, *CRYGC*, and *CRYGD*—were screened for the presence of a disease-causing mutation. The *CRYGA*, *CRYGC*, and *CRYGD* genes were mutation free in all of the family members. However, *CRYGB* revealed two novel mutations in the affected family members, with an autosomal dominant mode of inheritance. The inheritance pattern of the *CRYGB* mutation extended to the grandparental generation, in which both affected individuals were found to have mutant *CRYGB* genes, resulting in two different phenotypes. These mutations included a heterozygous “C” deletion in exon 2 (g.167delC, Exon 2), which was found to be maternally inherited from the Egyptian grandmother, and a heterozygous “G” deletion in the first intron of the *CRYGB* gene (g.67delG, intron 1), which was found to be paternally inherited from the Kuwaiti grandfather. In addition, a novel trinucleotide polymorphism (g.20–22 GGT>AAA, Intron 1) was detected in the CRYBG gene of three of the family members.

**Figure 2:**
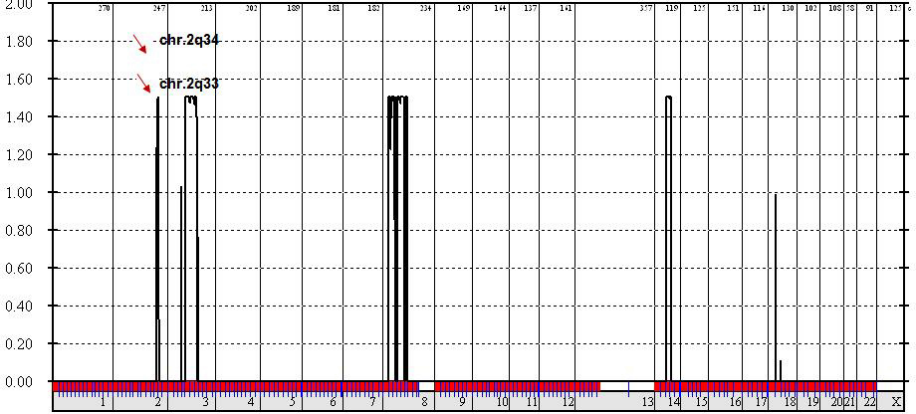
Whole genome linkage analysis. The results show the highest logarithm of odds (LOD) score regions, Chr.2q34 and Chr.2q33, spanning the crystalline beta A2 (*CRYGB A2*) and crystallin gamma (*CRYG*) gene cluster.

The heterozygous g.167delC, Exon 2 mutation (numbered starting from the first ATG codon) was detected in two of the affected family members (I-1 and III-2) who had anterior polar cataracts. These two patients were characterized by the presence of cataracts in the pupillary region (Figure 2).

The second novel heterozygous g.67delG, Intron 1 mutation (numbered starting from the first ATG codon) was detected in four of the affected family members (I-2, II-5, III-1, and III-4). Clinical and morphological results confirmed the presence of lamellar cataracts with complete penetrance in all of the patients with the g.67delG, Intron 1 mutation. An infant (III-3) who was found to have the same mutation has not yet shown any characteristic symptoms of cataracts, most likely due to the age factor. Another patient diagnosed with complete cataracts (II-3) was found to be a compound heterozygote with both g.167delC, Exon 2 and g.67delG, Intron 1. Neither of these mutations was detected in 50 healthy subjects, including the unaffected family members.

This study is the first to report three novel adjacent SNPs (g.20–22 GGT>AAA, Intron 1) next to the 3′ donor splice site of the CRYGB gene. This polymorphism was found to be maternally inherited (II-2) by the two affected daughters with lamellar (III-1) and anterior polar cataracts (III-2), whereas it was not detected in the third sibling (III-3) or in the 50 unrelated healthy subjects. The functional role of this polymorphism is not yet known.

## Discussion

Congenital cataracts are a major cause of eye abnormalities and frequently cause blindness in infants. Mutations in crystallin genes are reported to be the most common causes of congenital cataracts in humans and mice. Intensive research over many years has led to several new gene mutation detection techniques, of which microarray is regarded as an efficient alternative to molecular studies of inherited disorders. In this study, the Affymetrix Gene Chip Human Mapping 250 K microarray followed by cloning and sequencing was used to detect causative mutations resulting in three distinct phenotypes in a Kuwaiti family across three generations. Two novel mutations, a heterozygous g.167delC mutation in Exon 2 and a g.67delG mutation in Intron 1, were detected in the *CRYGB* gene of affected family members. The genotype and resulting phenotypic variations are well illustrated in our study. In addition, a novel trinucleotide polymorphism, g.20–22 GGT>AAA in Intron 1, was detected in this study.

The mammalian *CRYGB* gene consists of three exons and encodes a protein of 175 amino acids. The first exon encodes three amino acids, and the subsequent two exons each encode two Greek key motifs. The structural characteristics of the *CRYGB* protein account for its stability and solubility. It consists of numerous charged and polar side chains, many of which are organized in pairs or chains that form ionic and polar interactions. Changes in the amino acid sequences of crystallins may disrupt their normal structure in the lens and cause cataracts. The heterozygous C deletion in Exon 2 (g.167delC, Exon 2), which was detected in two of the affected family members (I-1 and III-2) with anterior polar cataracts, results in a truncated protein of 43 amino acids. The mutant protein is expected to contain the first 24 amino acids of the wild-type protein, and the subsequent deletion results in a frame shift, generating 19 nonconserved amino acids before translation termination at position 130. The binding of mutant γB crystallins to either of the α crystallin subunits may interfere with subunit exchange, which is regarded as a key step in preventing the formation of light-scattering aggregates of crystallins. Furthermore, the alignment of wild-type and mutant protein sequences shows that the proline at position 24 is conserved in both cases. In the wild-type protein, this proline is encoded by the CCC codon, while in the mutant, it is encoded by the CCA codon.

The second novel mutation, a heterozygous G deletion in Intron 1 (g.67delG, Intron 1), was detected in four of the affected family members with lamellar cataracts (I-2, II-5, III-1, III-3, and III-4). The specific molecular process that leads to the phenotypic manifestations of lamellar cataracts in patients with the g.67delG, Intron 1 mutation is not known. Because the mutation is located 27 bp from the splice acceptor site of the direct strand (5ꞌ-CAT TTT TCA G ^ ATC ACC TTC T-3ꞌ, confidence 0.95), this change might trigger an abnormal splicing spectrum, such as the differential inclusion or exclusion of regions of pre–mRNAs. Several studies have previously implicated intronic mutations in cataractogenesis. Studies of heat shock transcription factor 4 have revealed two intronic mutations (c.1020–25G>A/ c.1256+25C>T) in patients with age-related cataracts [19]. More recently, a splice site mutation segregating with familial cataract was reported in the first base of Intron 3 of the CRYBA1/A3 gene [20]. Another important role of introns that is currently under investigation is the transcription of the introns to small regulatory RNAs, such as microRNAs, that can regulate gene expression. However, these studies could not be performed in this family because all of the affected family members (except I-1) underwent surgery at an early age, and their lens tissues were not preserved. Another patient (II-3) with complete cataracts was found to be a compound heterozygote with both of the above mentioned mutations (g.167delC, Exon 2 and g.67delG, Intron 1). This patient received defective alleles from both of his parents, resulting in a new phenotype.

In this study, two different mutations in the *CRYGB* gene resulting in three distinct phenotypes were detected. The phenomenon of allelic heterogeneity, in which different mutations within the same gene produce different cataract phenotypes, is not new. Variations in phenotypic expression based on mutation location are clearly observed for the connexin gene (Cx50; GJA8), in which mutations may lead to pulverulent or jellyfish-like cataracts [21,22]. Allelic heterogeneity also accounts for the two different cataract phenotypes, nuclear and coralliform cataracts, that may be caused by mutations in the *CRYGD* gene [23,24]. These mutations may either interfere with the regulation of the protein or its ability to bind to other lens proteins. Further studies should be performed to explore the specific cellular and molecular processes that lead to the manifestation of different cataract phenotypes.

This study is the first to report three novel adjacent SNPs (g.20–22 GGT>AAA, Intron 1) in the CRYGB gene. The functional role of this trinucleotide polymorphism is not known. It was found to be maternally inherited in two affected daughters (III-1 and III-2), while the mother (II-2), who was a carrier, showed no obvious eye abnormalities. Therefore, this polymorphism is considered unlikely to be involved in cataractogenesis [25,26].

In conclusion, we report the linkage of ADCC to chromosome 2q33–37, which spans the *CRYGB* gene. This study is the first to report complex heterogeneous mutations in the *CRYGB* gene resulting in ADCC with three distinct phenotypes (lamellar, anterior polar, and complete cataracts) in the same family.
